# The Women’s Dental Association of the United States

**Published:** 1892-09

**Authors:** 


### THE WOMEN'S DENTAL ASSOCIATION OF THE UNITED STATES.

To the Editor:

Sir,—It is with great pleasure that we announce the organization of the Women's Dental Association of the United States. Not many years have passed (twenty-three) since the first woman graduated at an American dental college. To-day the profession recognizes, among its ablest practitioners, women graduates in dentistry. So general, indeed, has the practice of this profession by women become, in the leading cities of this country, that the formation of this Association may be regarded as but a natural sequence.

Among the leading dentists of this and other cities included in the new organization are Drs. Edith L. Brown, Elizabeth A. Davis, Annie T. Focht, B. M. Jarrett, Maria Lasser, Anna K. Letenmeier, Hannah M. Miller, Martha B. Moore, Mary H. Stilwell, Margaret Taylor, W. W. Wyeth, and Eliza Yerkes, of Philadelphia; Drs. A. Ireland and Olga Neymann, of New York; Dr. F. E. Hoopes, of Baltimore; Dr. Edith Jewell, of Washington, D. C.; Dr. A. F. Reynolds, of Boston; and Dr. E. L. Benham, of Chicago.

A constitution and by-laws have been adopted, and a charter is about to be applied for. The object of the Association is the advancement of the professional standard, the mutual study of important cases, and especially the support and encouragement which must necessarily result from membership of such a body.

The President is Dr. Mary H. Stilwell; Vice-President, Dr. Anna K. Letenmeier; Recording Secretary, Dr. Eliza Yerkes; Corresponding Secretary, Dr. A. T. Focht; Treasurer, Dr. Maria Lasser; Executive Committee, Drs. Elizabeth A. Davis, Bertha M. Jarrett, and Hannah M. Miller.

Active members are elected from among graduates of dental colleges who are of good standing and in active practice. Names of proposed new members are submitted to the Recording Secretary, whose address is 1404 Chestnut Street, Philadelphia, and who reports them to the Executive Committee. Upon approval by this committee they are submitted to a vote of the Association, and a two-thirds vote secures election. There is an initiation fee of one dollar. The annual dues are also one dollar.

In addition to the active members, the constitution provides for the election, as associate members, of those who have rendered eminent service to the profession.

It is difficult to speak in terms of too high praise of this important movement. While it is customary for men to organize for all purposes in which union gives strength, and while dental associations of men are numerous, women dentists have heretofore stood alone, or, if connected with existing dental societies, have figured mainly as exceptions to the average. The character of those connected with the Women's Dental Association gives assurance that it cannot but elevate the standard of the profession and strengthen the position of its members, as dentists, in the community. Its code of ethics, rightly interpreted, will add dignity to their calling and tend to the establishment of mutually beneficial relations.

It is more than probable that in a short time the influence of this Association will be felt for good, not only in Philadelphia, where it originated, but to a greater or less extent throughout all the United States.

